# Recognize the Significance of Detecting Toxic Granules in Sepsis

**DOI:** 10.14789/ejmj.JMJ25-0025-P

**Published:** 2025-10-24

**Authors:** SHOUICHI SATO

**Affiliations:** 1 Clinical Engineering, Faculty of Medical Science, Juntend University, Chiba, Japan; 1 Clinical Engineering, Faculty of Medical Science, Juntendo University, Chiba, Japan

**Keywords:** toxic granule, neutrophil, sepsis, blood smear, immune response

## Abstract

Recent studies have reviewed the significance of recognizing toxic granulation in neutrophils, a critical morphological change in sepsis, reflecting the immune system’s acute response to infection. Toxic granules, dark cytoplasmic granules, arise from accelerated neutrophil maturation under cytokine stimulation, especially granulocyte colony-stimulating factor (G-CSF). Their presence correlates with disease severity and inflammatory markers like C-reactive protein (CRP) and procalcitonin. Toxic granules often appear alongside Döhle bodies and cytoplasmic vacuolization, forming a broader “toxic change” profile. Persistent or severe toxic granulation may indicate a poor prognosis. While commonly associated with sepsis, similar features can occur with G-CSF therapy or bone marrow recovery, highlighting the need for contextual interpretation. Efforts are underway to standardize grading and integrate automated image analysis to improve diagnostic value. Toxic granulation remains a simple yet useful tool for early sepsis recognition, especially when combined with clinical and laboratory data. It reinforces the continued relevance of peripheral blood smear analysis in modern diagnostics.

Recent investigations into toxic granulation in the context of sepsis have advanced our understanding of the morphological and functional changes occurring in neutrophils during systemic infection. Toxic granulation refers to the presence of dark, coarse granules in the cytoplasm of neutrophils, which are not typically found in mature cells under normal physiological conditions. These granules are the result of an accelerated granulopoiesis and incomplete maturation, typically in response to inflammatory stress, particularly in sepsis. Although toxic granules have long been recognized as a hallmark of severe infection, new evidence is refining their diagnostic and prognostic utility in the era of sepsis research.

Sepsis is characterized by a dysregulated immune response to infection, which involves the activation of the innate immune system and neutrophil mobilization. One of the earliest morphologic responses of neutrophils in sepsis is the emergence of toxic granules. These granules, composed primarily of primary granule contents like myeloperoxidase and other lysosomal enzymes, reflect the neutrophil’s heightened antimicrobial activity. Unlike secondary granules that appear in mature neutrophils under homeostasis, these toxic granules suggest that the neutrophils were released prematurely from the bone marrow. Recent studies, particularly those examining peripheral blood smears of septic patients, have provided insights into the temporal dynamics of toxic granule appearance and its correlation with clinical severity. Subhash et al.^1)^ analyzed neutrophil morphology in confirmed sepsis patients and found a high prevalence of toxic granulation in the early phase of infection. Notably, they reported that the degree of toxic granulation positively correlated with C-reactive protein (CRP), procalcitonin, and Sequential Organ Failure Assessment (SOFA) scores^2)^. This supports the notion that toxic granules are not only markers of infection but also reflect disease severity.

Moreover, toxic granulation is often accompanied by other morphological features such as Döhle bodies, light blue cytoplasmic inclusions composed of rough endoplasmic reticulum, and cytoplasmic vacuolization^3)^. These changes, when present together, form a broader picture of "toxic change" in neutrophils. These morphological hallmarks have been proposed as practical tools for early sepsis recognition in emergency and critical care settings. Studies have suggested that incorporating neutrophil morphology assessment into routine blood smear evaluation could enhance early sepsis detection, particularly when laboratory biomarkers are pending or inconclusive^3)^.

The development of toxic granules is known to be a response to cytokine stimulation, most notably granulocyte colony-stimulating factor (G-CSF), which is elevated in response to infection. G-CSF drives the proliferation and rapid maturation of myeloid precursors, resulting in the release of immature or partially matured neutrophils with retained primary granules. This explains why toxic granulation is frequently observed alongside a “left shift” in the white blood cell differential, indicating an increase in band forms and other immature myeloid cells. Such hematological profiles are commonly reported in septic patients^4)^. From a prognostic perspective, recent findings suggest that persistent or severe toxic granulation may be associated with worse outcomes in sepsis. This implies that while early toxic granulation may represent a robust neutrophilic response to infection, prolonged or excessive expression may reflect ongoing systemic inflammation and poor immunological resolution^5)^. There is also growing interest in the standardization of grading systems for toxic granulation. Currently, its assessment is largely subjective, depending on the experience and judgment of laboratory personnel examining peripheral blood smears. A few recent initiatives have proposed semi-quantitative scoring methods to categorize the severity of toxic granulation (e.g., mild, moderate, severe) based on the number of affected neutrophils and granule density. These scoring systems, when integrated with other clinical markers, have the potential to improve diagnostic precision and allow for risk stratification in septic patients^6)^.

Another area of research is the differentiation between infection-induced toxic granulation and that induced by non-infectious causes. For instance, patients receiving G-CSF therapy or undergoing recovery from bone marrow suppression (e.g., after chemotherapy) may show similar morphological changes. This necessitates cautious interpretation of toxic granules in the clinical context. However, the co-existence of clinical symptoms, elevated infection markers, and other neutrophilic abnormalities can help distinguish sepsis from such mimics^4)^.

Advances in digital hematopathology and machine learning are also contributing to the understanding of toxic granulation. Automated image analysis systems are being trained to recognize and quantify toxic changes in white blood cells, including granulation patterns. Such tools may reduce observer variability and provide real-time morphological analysis in critical care settings. While still under development, these technologies hold promise for incorporating morphological diagnostics into automated sepsis screening protocols^7, 8)^.

Finally, the presence of toxic granulation underscores the importance of not disregarding the peripheral blood smear in modern clinical practice. In an age dominated by molecular diagnostics and high-throughput lab tests, the humble blood smear continues to provide valuable insights. The morphological clues it offers, including toxic granulation, serve as real-time reflections of the bone marrow’s response to systemic inflammation and infection. They are especially valuable in resource-limited settings or in early phases of sepsis when molecular confirmation is delayed^1, 9, 10)^.

Future research focused on standardization, automation, and integration with machine learning may elevate toxic granule assessment from a descriptive observation to a quantitative and actionable biomarker in sepsis care. For example, Zhang et al.^10)^ introduced a method to quantitatively assess the presence and extent of toxic granules in neutrophils using a hematology analyzer that can measure leukocyte volume and cellular complexity. The parameters are associated with more granules in the cytoplasm and stronger staining, making the evaluation of toxic granules more objective and reproducible. Similarly, another specific hematology analyzer, which can also count toxic granules of neutrophils, enables the evaluation of granulation on a 24-hour basis because of a simple and easy method^11)^. The popularization of these machines will make evaluating toxic granulation routine work.

In summary, toxic granulation remains a clinically significant morphological change in sepsis. Recent evidence supports its role as an early marker of systemic infection, a potential indicator of severity, and a prognostic factor. Integrating its evaluation into routine diagnostic workflows, alongside clinical and biochemical parameters, can enhance sepsis detection and monitoring.

**Figure 1:**
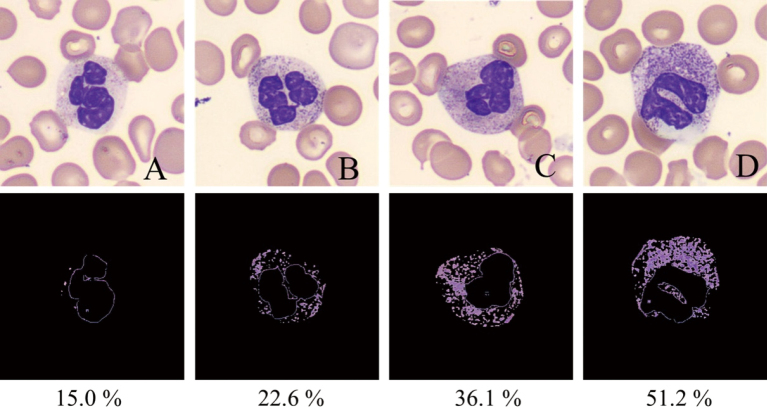
Representative images of neutrophils with varying degrees of toxic granulation and their corresponding segmented images used for granule quantification Upper panels (A-D) present original microscopic images of neutrophils stained with May-Grünwald-Giemsa, showing increasing granule intensity from left to right. Lower panels display the corresponding segmented images processed using OpenCV, an opensource computer vision library commonly used for image analysis. In this study, OpenCV was employed to extract cytoplasmic granules through intensity-based thresholding and contour detection. The granule regions were binarized and quantified. Granule_Percentage, defined as the ratio of the extracted granule area to the total cytoplasmic area, was used as an objective index to assess the severity of toxic granulation. The numerical values shown beneath each segmented image represent the calculated Granule_Percentage for the corresponding neutrophil.

## Author contributions

SS wrote and reviewed the manuscript and approved the final version.

## Conflicts of interest statement

The author declare that there are no conflicts of interest.
